# Data in support of NFκB and JNK pathways involvement in TLR3-mediated HIV-1 transactivation, expression of IL-6 and transcription factors associated with HIV-1 replication

**DOI:** 10.1016/j.dib.2015.12.022

**Published:** 2015-12-17

**Authors:** Biju Bhargavan, Shawna M. Woollard, Georgette D. Kanmogne

**Affiliations:** Department of Pharmacology and Experimental Neuroscience, University of Nebraska Medical Center, Omaha, NE 68198-5800, USA

**Keywords:** TLR3, HIV transactivation, transcription factors, IL-6, human macrophages

## Abstract

In the present article, using human monocyte-derived macrophages and cell lines containing integrated copies of the HIV-1 promoter, we show the effects of TLR3 ligands on the pro-inflammatory cytokine IL-6. We further show the effects of TLR3 ligands on HIV-1 transactivation and transcription factors involved in HIV-1 replication. This article complements the data reported by the authors, “Toll-Like receptor-3 mediates HIV-1 transactivation via NFκB and JNK pathways, and histone acetylation, but prolonged activation suppresses Tat and HIV-1 replication” (Bhargavan et al., 2015) [1], and the interpretation of these data can be found in the research article published by the authors in Cellular Signaling in 2015 (Bhargavan et al., 2015) [1].

## Specifications Table

**Table:** 

Subject area	Biology
More specific subject area	Molecular biology, Virology
Type of data	Graph, Figure
How data was acquired	Cell culture, Real-time PCR, Luciferase assay, CAT-ELISA
Data format	Analyzed
Experimental factors	Human monocyte-derived macrophages and cell lines containing integrated copies of the HIV-1 promoter; HIV-1 infection; treatment with TLR3 ligands, and pharmacological inhibitors
Experimental features	Real-time PCR, luciferase assay, and chloramphenicol acetyltransferase assay
Data source location	University of Nebraska Medical Center, Omaha, USA
Data accessibility	Data are with this article

## Value of the data

• The approach used in this data article allows for the prediction of biological pathways involved in receptor–ligand interactions.
• Data on the mechanistic basis of TLR3-HIV interactions would be useful to others investigating viral transactivation and replication.
• Others interested in studying the functional effects of receptor–ligand interactions could learn from our approach.
• This approach would be of interest when determining the critical role of time on gene transcription and expression.

## 1 Data

To determine whether the TLR3 ligand polyinosinic-polycytidylic acid (PIC) can interact with HIV-1 to affect gene transcriptional regulation in human monocyte-derived macrophages (MDM), time-dependent and concentration-dependent real-time PCR analyses were performed on uninfected or HIV-1 infected human MDM treated with PIC. Genes analyzed included the pro-inflammatory cytokine IL-6 and transcription factors known to regulate the HIV-1 promoter activity (STAT-1, REL-B, JUN, CEBPA, and CEBPG). To investigate the role and involvement of these transcription factors in TRL3-mediated HIV-1 transactivation, pharmacological inhibitors targeting their signaling pathways were used, with and without TLR3 ligands, in luciferase and chloramphenicol acetyltransferase (CAT) ELISA assays on TZM-bl and U38 cells, both of which contained integrated copies of the HIV-1 promoter.

### 2 Experimental design, materials and methods

#### 2.1 HIV-1 infection of MDM and real-time PCR

**Fig. 1:**
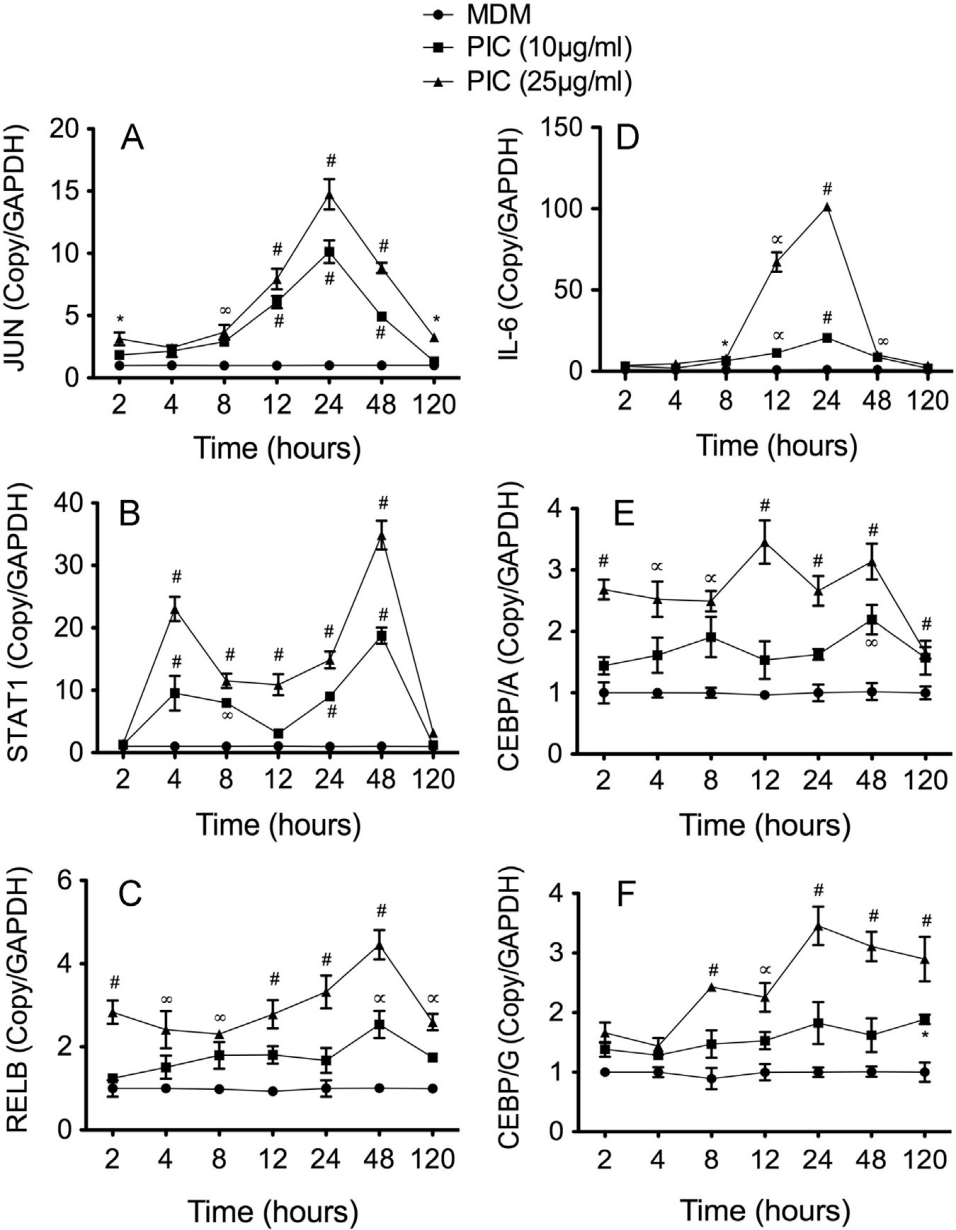
Real-time PCR quantification of JUN (A), STAT1 (B), RELB (C), IL-6 (D), CEBPA (E), and CEBPG (F) mRNA in untreated and human macrophages treated with the TLR3 ligand PIC. For both MDM treated with 10 μg/ml and 25 μg/ml PIC, *P<0.05, ∞*P*<0.001. ∝*P*<0.001, #*P*<0.0001, compared to untreated MDM.

**Fig. 2:**
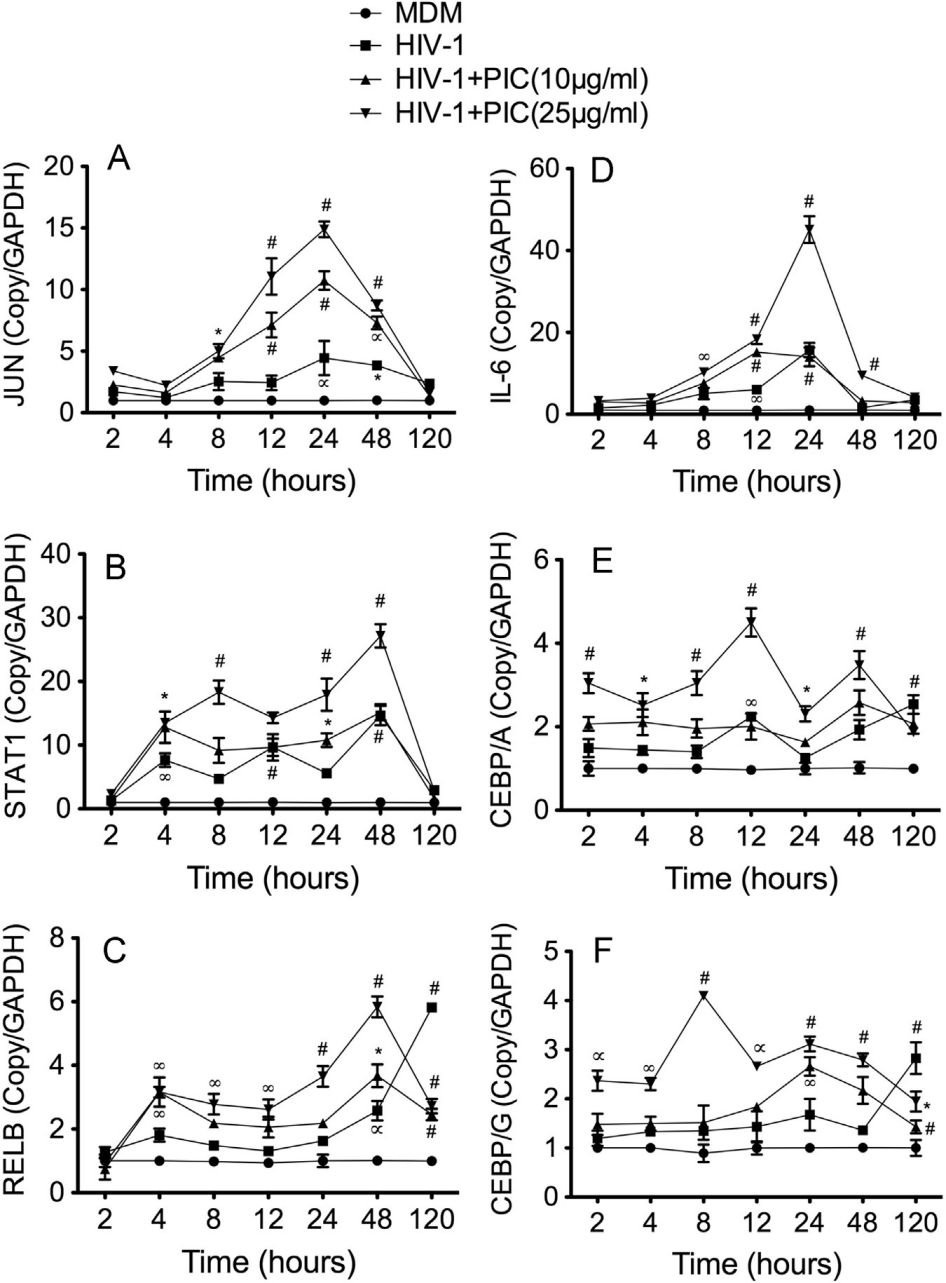
Real-time PCR quantification of JUN (A), STAT1 (B), RELB (C), IL-6 (D), CEBPA (E), and CEBPG (F) mRNA in HIV-1-infected human macrophages, untreated or treated with the TLR3 ligand PIC. *P<0.05, ∞*P*<0.001. ∝*P*<0.001, #*P*<0.0001. *P*-values of infected MDM (HIV-1) are in comparison to non-infected controls (MDM), and *P*-values of infected MDM treated with PIC (HIV-1+PIC(10 μg/ml), and HIV-1+PIC(25 μg/ml)) are in comparison to infected MDM.

Human MDM were obtained from freshly elutriated human monocytes as previously described [1], [2], cultured in 6-well plates (2 million cells per well), and treated with 10 or 25 μg/ml PIC for 2–120 h, with each experimental condition performed in triplicate. In separate experiments, MDM were infected with HIV-1_ADA_ at a multiplicity of infection of 0.01 as previously described [1], [2], [3], [4], with or without PIC treatment (10 and 25 µg/ml) for 2–120 h, with each experimental condition performed in triplicate. Following treatment, cells were harvested, total RNA was extracted using the Trizol reagent, and real-time PCR performed as described in the main manuscript [1]. All PCR reagents, probes and primers were from Applied Biosystems and primers IDs were as follows: CEBPA (Hs00269972_s1), CEBPG (Hs01922818_s1), JUN (Hs99999141_s1), STAT1 (Hs00234829_m1), RELB (Hs00232399_m1), IL-6 (Hs00985639), and GAPDH (Hs99999905_m1). For each gene and each sample, data was normalized to the sample’s GAPDH to quantify the effects of PIC (10 and 25 μg/ml) on IL-6, STAT-1, REL-B, JUN, CEBPA, and CEBPG mRNA in uninfected (Fig. 1) and HIV-1-infected (Fig. 2) human MDM.

#### 2.2 Luciferase and chloramphenicol acetyltransferase (CAT) assays

**Fig. 3:**
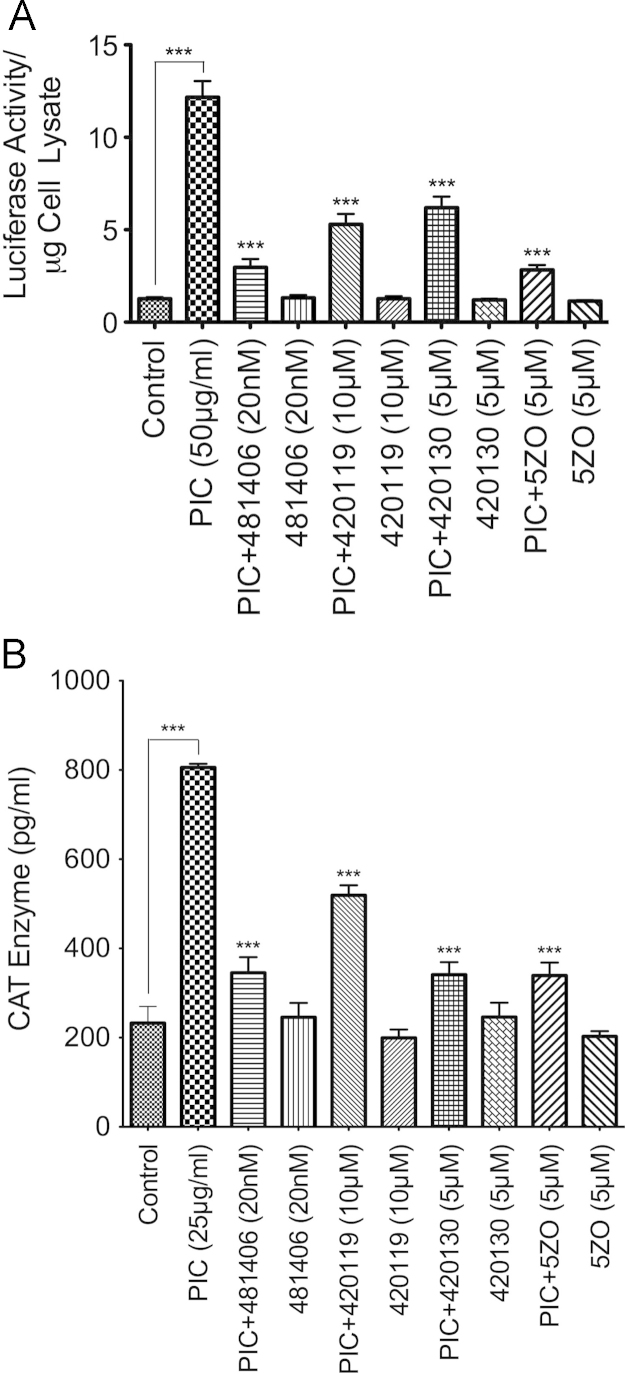
Quantification of HIV-1 transactivation in TZM-bl (A) and U38 (B) cells treated with TLR3 ligands, with or without the inhibitor of NFκB transcriptional activation ( 481406), the JNK inhibitor ( 420119), the inhibitor of c-Jun/JNK complex ( 420130), and the MEKK7/MKK7 inhibitor (5ZO). ***P<0.001; *P*-values for inhibitors-treated samples are in comparison to the HIV-1 promoter activity in PIC-treated cells.

TZM-bl and U38 cells were treated for 48 h with PIC (25 or 50 µg/ml), with or without the inhibitor of NFκB transcriptional activation (481406, 20 nM), the JNK inhibitor ( 420119, 10 µM), the inhibitor of c-Jun/JNK complex (420130, 5 µM), and the MEKK7/MKK7 inhibitor (5ZO, 5 µM). Each experimental condition was performed in triplicate and following treatment, cells were harvested, washed with phosphate-buffered saline, and lysed as described [1]. Cell lysates were then used to quantify the luciferase activity (Fig. 3A) and the CAT activity (Fig. 3B) in each sample using the Luciferase Assay System (Promega, Madison, WI) and the CAT ELISA kit (Roche Diagnostics Indianapolis, IN), as described in the main manuscript [1].

**Fig. 4:**
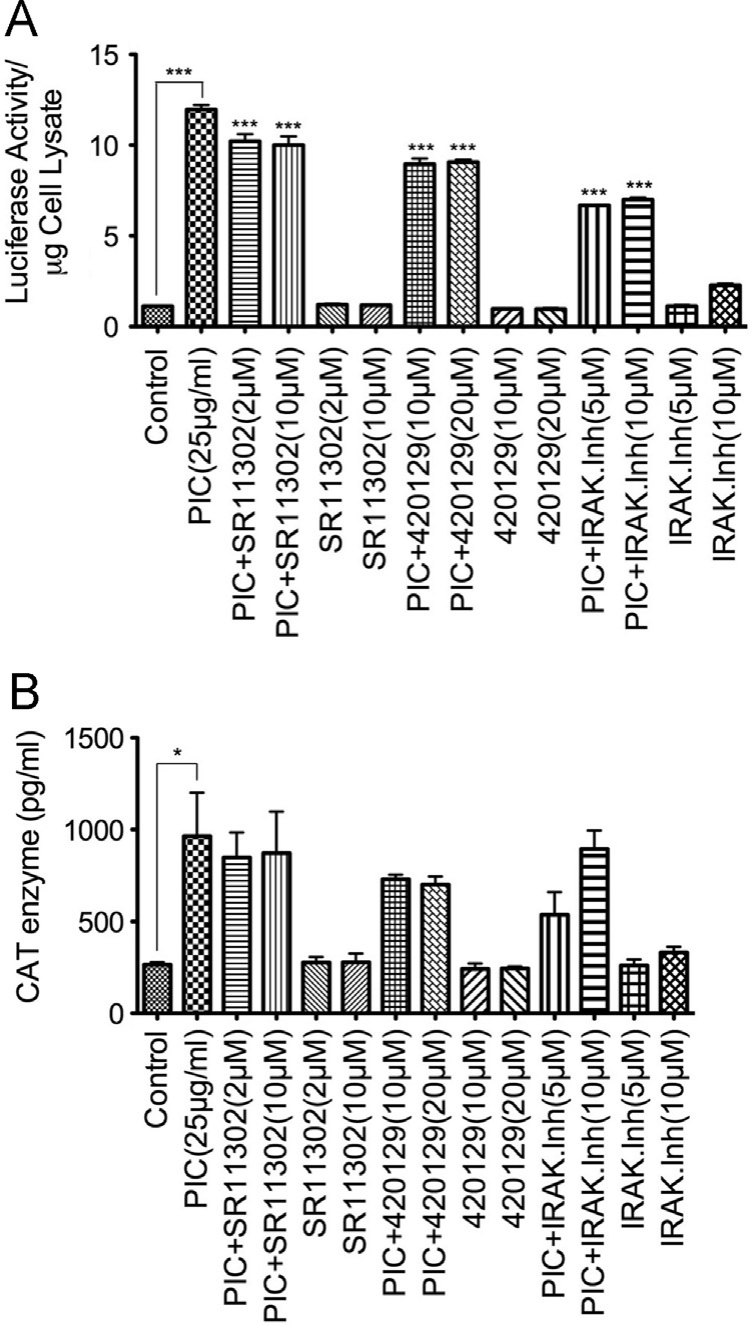
Quantification of HIV-1 transactivation in TZM-bl (A) and U38 (B) cells treated with TLR3 ligands, with or without the inhibitor of AP-1 transcriptional activity (SR11302), the ATP-competitive inhibitor of JNK (420129), and the IRAK1/4 inhibitor ***P<0.001; *P*-values for inhibitors-treated samples are in comparison to the HIV-1 promoter activity in PIC-treated cells.

In separate experiments, cells were treated for 48 h with PIC (25 µg/ml), with or without the AP-1 inhibitor (SR11302, 2 µM and 10 µM), the JNK inhibitor V (420129, 10 µM and 20 µM), and the IRAK-1/4 inhibitor (5 µM and 10 µM). Each experimental condition was performed in triplicate and following treatment, the effects of PIC and inhibitors on HIV-1 transactivation was quantified in TZM-bl cells (Fig. 4A) or U38 cells (Fig. 4B). The main manuscript [1] includes the manufacturer׳s names, catalog numbers, and mechanisms of action of each inhibitor.
